# Letter from Willis G. Edwards, M. D., to Prof. Brainard

**Published:** 1849-01

**Authors:** 


### ARTICLE IX.

*Letter from* WILLIS G. EDWARDS, M. D., *to Prof. Brainard.*

[The following interesting letter is from a highly intelligent and talented physician of Alton in this state, who is pursuing his studies in the Parisian Hospitals. We hope to receive from him, occasionally, an account of medical matters as they occur in Europe, to lay before our readers.]

*Paris, Oct.* 21, 1848.

DEAR SIR:—Some time has passed since I saw you in New York, and promised to communicate to you the address of the Parisian Medical Society. It would have given me much pleasure to have done so at an earlier day, but it is only within the last week that I have been enabled to learn the place of its meetings. The unsettled condition of political matters existing here has driven most of the foreign residents from the city, including many members of the society; and, in fact, the number left was so small that much difficulty has been experienced in keeping up the meetings, and, during the vacations, they have, as usual, been entirely suspended. Several young Americans, who are pursuing their studies here, are active members, and sustained the meetings for some time almost alone. It ought to be a gratifying reflection to the American medical profession that there exists, in the great centre of medical knowledge of the world, an association of its own members having for its object their mutual improvement and the general advancement of the science—it *certainly* is an evidence of their enterprize and enthusiasm. The society rooms are at No. 3, Rue Racine.

Presuming that you will like to hear something of what is at present interesting the profession here, I will fill my sheet with a very brief sketch of the first impressions which I received on visiting the hospitals, and a brief statement of a few of the most interesting cases that have occurred in them since my arrival. The bloody Revolution of June filled the hospitals with a most interesting class of patients, and presented a rare opportunity to those who were fortunate enough to be here at the time, for the study of gunshot wounds. On my arrival, two weeks afterwards, though the primary operations were over, and many of the worst cases had terminated, I found the hospitals crowded, and have had a good opportunity of observing the practice adopted by the different distinguished professors. My first visit was to the wards of M. Jobert, at the Hospital Saint Louis, where my attention was particularly attracted by his novel mode of treating fractures, which consists, as you probably know, in simple extension without the application of splints or bandages. The numerous cases of fractures of all characters in his wards gave a fine opportunity for testing the merits of his treatment, and I must say, that so far as my observations extended, the results were more favorable than my previous impressions had led me to anticipate. On an average, his cures were as perfect, and followed by as little deformity or shortening of the limb, as where the ordinary apparatus was employed. In cases of compound fracture, this mode possesses the advantage of affording a free escape to the abundant discharges attending cases of this character, and renders the treatment of the wound more easy and efficient. In the wards of Velpeau, Roux, and Blandin, the ordinary treatment of fractures is adopted, combined with excessive quantities of dressing to the wound of the soft part, in compound cases, which cause the limb to swell to an enormous size, and to present on their removal a perfect pool of pus, in which the whole limb is bathed, and by which the parts are so discolored that their real condition is discovered with difficulty. The universal practice here is to load wounds of all descriptions with most excessive quantities of charpie, bandages, &c., and the antipathy to union by first intention seems to be universal. I have seen but one single case among 47 amputations where this result was secured or rather where it was permitted. Wherever you find amputations you will find discolored, suppurating, and often offensive stumps. On the whole, the treatment of gunshot wounds adopted here, has not impressed me favorably, and though there are many surprising recoveries, the general results have not been what I had expected from French surgery.

A statistical account will probably be published, embracing all the interesting cases which have occurred, and will probably furnish much valuable information on the comparative mortality from wounds of different regions. It is difficult to follow a sufficient number of cases to arrive at any positive results, unless one is connected with the hospital service; but, judging from all the information which I have been able to gain, wounds of the chest, after the mortality of the first few days, have been less fatal than those of the thigh, involving the femur. My impressions, in regard to the fatality of the former, have been considerably modified by what I have seen here. In a number of instances where the ball had traversed a portion of the lungs the patients have recovered, and in a still greater number where the ball had entered some portion of the chest, and the diagnosis was doubtful.

A case of wound of the brain occurred at La Pitie, possessing much interest, both on account of the time to which the life of the patient was protracted, and the physiological phenomenon which it presented. I saw the patient three weeks after he received the injury. He was slightly comatose; respiration slow; skin cool and moist,and exhibiting signs of capillary congestion; pulse slow, feeble, and compressible; pupil contracted, and partially insensible, &c. I learned that his symptoms had undergone a considerable change for the worse during the preceding twenty-four hours, and supposed that the coma would terminate in death. He was, however, bled and purged freely, and within a few days was about, entirely relieved of the urgent symptoms. The wound was situated in the anterior and superior portion of the right parietal bone, and passed backwards, and a little inwards and downwards. It was suppurating, and contained a dirty looking slough. The motion of the brain was distinctly visible, and consisted in a *double movement*, the first and most forcible being synchronous with the contraction of the heart, and the *latter* with the *respiration*. The patient continued to improve up to the fourth week from the time that I first saw him, and, until a few hours of his death, seemed to be recovering. The particulars of the post mortem I could not learn, but understood that the ball was near the base of the skull, having penetrated the substance of the brain to about the depth of 2½ inches, and afterwards gravitated to the position which it occupied.

